# Thymol@Natural Zeolite Nanohybrids for Chitosan/Polyvinyl-Alcohol-Based Hydrogels Applied as Active Pads

**DOI:** 10.3390/gels9070570

**Published:** 2023-07-12

**Authors:** Constantinos E. Salmas, Eleni Kollia, Learda Avdylaj, Anna Kopsacheili, Konstantinos Zaharioudakis, Stavros Georgopoulos, Areti Leontiou, Katerina Katerinopoulou, George Kehayias, Anastasios Karakassides, Charalampos Proestos, Aris E. Giannakas

**Affiliations:** 1 Department of Material Science and Engineering, University of Ioannina, 45110 Ioannina, Greece; tasos.karakassides@gmail.com; 2 Laboratory of Food Chemistry, Department of Chemistry, National and Kapodistrian University of Athens, Zografou, 15771 Athens, Greece; elenikollia@chem.uoa.gr (E.K.); leardaavdy@chem.uoa.gr (L.A.); akopsacheili@chem.uoa.gr (A.K.); 3 Department of Food Science and Technology, University of Patras, 30100 Agrinio, Greece; zacharioudakis.k@upatras.gr (K.Z.); sgeorgop@upatras.gr (S.G.); aleontiu@upatras.gr (A.L.); akaterin@upatras.gr (K.K.); gkechagi@upatras.gr (G.K.)

**Keywords:** chitosan, polyvinyl-alcohol, natural zeolite, thymol, active packaging, antibacterial activity, strawberry preservation

## Abstract

Currently, food saving, a circular economy, and zero environmental fingerprints are of major interest. Scientific efforts for enhanced food preservation using “green” methods have been intensified. Even though chemicals could achieve such targets effectively, the global trend against the “greenhouse effect” suggests the use of environmentally friendly biobased materials for this purpose. In this study, the promising biopolymer chitosan is incorporated with the promising biodegradable polymer polyvinyl alcohol to produce an improved biopolymeric matrix. This biodegradable biopolymer was further mixed homogeneously with 15% thymol/nano-zeolite nanohybrid material. The properties of the final developed film were improved compared to the relevant values of chitosan/polyvinyl alcohol film. The mechanical properties were enhanced significantly, i.e., there was a 34% increase in Young’s modulus and a 4.5% increase in the ultimate tensile strength, while the antioxidant activity increased by 53.4%. The antibacterial activity increased by 134% for *Escherichia coli*, 87.5% for *Staphylococcus aureus*, 32% for *Listeria monocytogenes*, and 9% for *Salmonella enterica*. The water vapor diffusion coefficient and the oxygen permeability coefficient decreased to −51% and −74%, respectively, and thus, the water vapor and oxygen barrier increased significantly. The active pads were used in strawberries, and the antimicrobial activity evaluation against the mold of fungi was carried out. The visual evaluation shows that the active pads could extend the shelf life duration of strawberries.

## 1. Introduction

Currently, the fruit and vegetable industries produce a large amount of waste due to overproduction and the short shelf life of such products [1]. Following the circular economy spirit, different strategies were proposed to reduce such waste. One of the dominant proposed practices is the increase in fruit and vegetable shelf life, and thus the extension of time that consumers can buy and consume fruits and vegetables [2]. This method can also help to keep the nutrition value of vegetables and fruits high [3,4]. The use of polysaccharide-based edible films and coatings as novel nontoxic, renewable, and degradable materials to preserve fruits and control their aerobic respiration is also a proposed method, which follows the sustainability and circular economy model [5,6]. Recent studies have shown that polysaccharide-based films and coating control and retard fruits’ and vegetables’ respiration rates. Furthermore, they provide the amount of water that is necessary for the preservation of such foods [7].

Cellulose, casein, zein, soy protein, and chitosan are some of the several possible edible coatings for fruits. A polysaccharide-based film must be odorless, tasteless, and transparent in order to be used as a fruit-based coating [8]. For such edible coatings, it is not easy to measure their gas permeation after being placed on fruits. For this purpose, separate flat films usually need to be prepared and tested. Chitosan (CS) has a great potential to be used as alternative film and coating in the future, as it is derived from the second most abundant biopolymer, the chitin [9,10]. CS is biodegradable, non-toxic, and has significant antibacterial properties against Gram-positive and Gram-negative bacteria [10]. CS also has excellent film-forming properties, good mechanical properties, and selective permeability to gasses such as CO_2_ and O_2_ [11,12]. Even though CS is generally recognized as safe (GRAS), it is still not allowed to be used as a food additive [13].

Polyvinyl alcohol (PVOH) is a water-soluble, biodegradable synthetic biopolymer that has been shown to have a great mixability and compatibility with CS to develop CS/PVOH food packaging films [14]. In addition, it was previously shown that PVOH can enhance the antibacterial activity of CS against various food pathogens [15,16].

Essential oils (EOs) are provided as alternative naturally abundant antioxidant and antibacterial components that can be used as sustainable food additives and food preservatives in the food packaging sector [17,18].

In the literature, there is a plethora of studies providing CS/EO-based films for food packaging films and coating applications [19]. A new technology for the use of EOs as novel food preservatives in polymer and biopolymer films and coatings provides control of their release by immobilizing them on naturally abundant adsorbents such as nanoclays [19,20,21,22]. Recently, zeolites were proposed as an alternative nano-reinforcement raw material for food packaging applications [23]. Natural zeolites (NZs) were also proposed as potential nanocarriers for EO controlled release in pork fillet food packaging films [24] and kiwi fruit coatings [16].

Although strawberry (*Fragaria ananassa*) is a fruit with a high nutritional value, it has a short shelf life due to water loss, texture softening, physiological deterioration, and microbiological decay [25,26,27]. In the literature, there are many studies with EO incorporated into CS-based films, which have been applied as coatings and succeeded in preserving and maintaining the quality of strawberries [28,29,30,31,32]. Based on the available literature, no reference has been found that reports a process where thymol (TO) is initially absorbed into NZ to create a TO@NZ nanohybrid that is dispersed within a CS/PVOH matrix once formed. This procedure produces active pads, which release TO in a controlled manner in the packaging environment, and this package was tested for the preservation of strawberries.

In this study, novel CS/PVOH-based films with NZ and NZ modified with thymol (TO@NZ) are developed via a solution casting method. The content of NZ and TO@NZ on CS/PVOH-based films varied between 5, 10, and 15 wt.%. The obtained CS/PVOH/xNZ and CS/PVOH/xTO@NZ films (x = 5, 10 and 15) were physiochemically characterized via XRD analysis and FTIR spectroscopy. In advance, the tensile, water–oxygen barrier, antioxidant, and antibacterial properties of such CS/PVOH/xNZ and CS/PVOH/xTO@NZ films were studied. The most active CS/PVOH/xNZ and CS/PVOH/xTO@NZ films were applied as active pads for strawberries. The following are the points of innovation of the current work: (i) CS/PVOH/xNZ and CS/PVOH/xTO@NZ active films are developed and characterized for the first time, and (ii) such CS/PVOH/xNZ and CS/PVOH/xTO@NZ films are applied as active pads for strawberries for the first time.

## 2. Results and Discussion

### 2.1. XRD Analysis of CS/PVOH/xNZ and CS/PVOH/xTO@NZ Films

**Figure 1:**
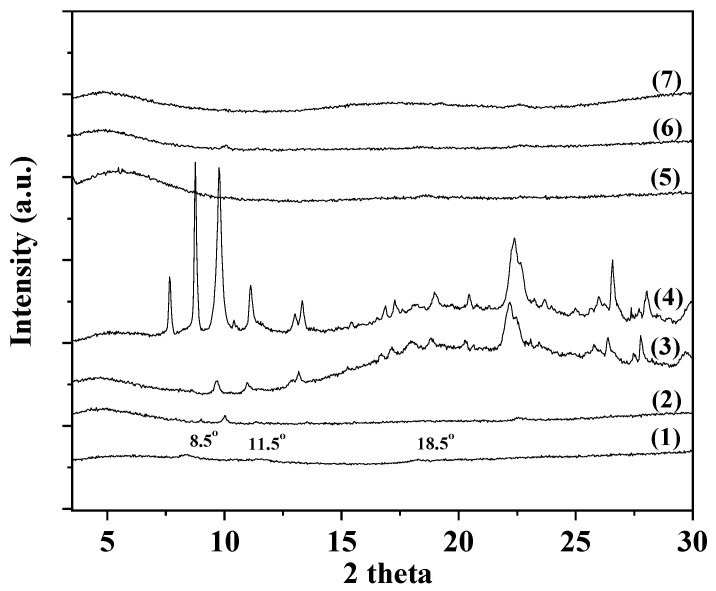
XRD plots of (1) CS/PVOH, (2) CS/PVOH/5NZ, (3) CS/PVOH/10NZ, (4) CS/PVOH/15NZ, (5) CS/PVOH/5TO@NZ, (6) CS/PVOH/10TO@NZ, and (7) CS/PVOH/15TO@NZ obtained films.

In Figure 1, the XRD plots of all obtained CS/PVOH/xNZ and CS/PVOH/xTO@NZ films as well as the pure CS/PVOH film are shown for comparison.

As it is observed in Figure 1 (see plot line (1)), the XRD plot of the pure CS/PVOH corresponds to an almost amorphous biopolymer structure. Only the broad reflections of CS at around 8.5°, 11.5°, and 18.5° 2 thetas are observed, which correspond with the hydrated crystal structure of CS [15,16]. In the case of the CS/PVOH/xNZ film plots (see plot lines (2), (3), and (4)), the characteristic peaks of NZ are observed, which are attributed to the Heulandite Ca(Si_7_Al_2_)O_16_ 6H_2_O monoclinic crystal phase (PDF-41-1357). As the wt.% content of NZ is increased, the characteristic peaks of NZ are further increased. This suggests a non-uniform dispersion of NZ in the CS/PVOH matrix, and that the aggregates of NZ are probably obtained. In the case of the CS/PVOH/xTO@NZ film plots (see plot lines (5), (6), and (7)), the characteristic peaks of NZ are not observed, suggesting a homogeneous dispersion of modified TO@NZ in the CS/PVOH matrix, supported by the TO molecules adsorbed in the NZ.

### 2.2. FTIR Spectroscopy of CS/PVOH/xNZ and CS/PVOH/xTO@NZ Films

**Figure 2:**
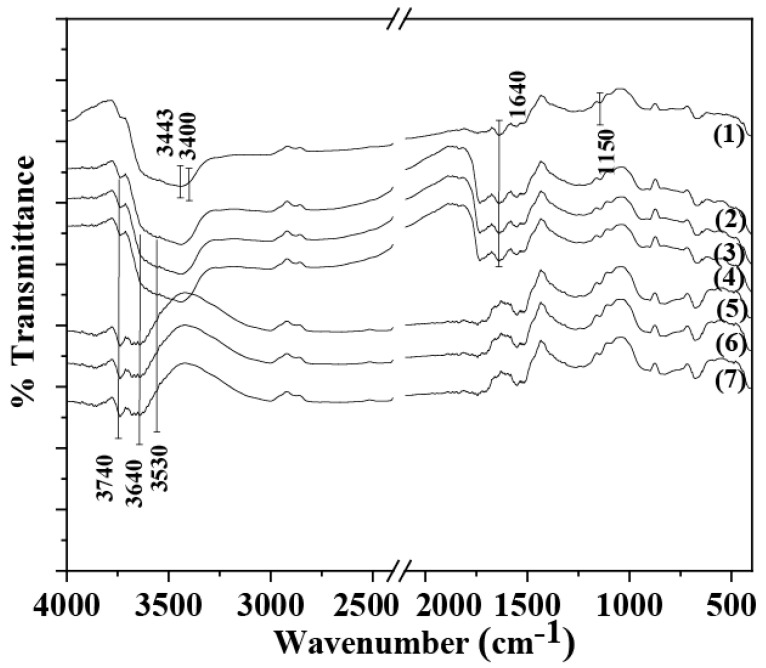
FTIR plots of (1) CS/PVOH, (2) CS/PVOH/5NZ, (3) CS/PVOH/10NZ, (4) CS/PVOH/15NZ, (5) CS/PVOH/5TO@NZ, (6) CS/PVOH/10TO@NZ, and (7) CS/PVOH/15TO@NZ obtained films.

In Figure 2, the FTIR spectra of the CS/PVOH/xNZ and CS/PVOH/xTO@NZ films as well as the pure CS/PVOH film are observed for comparison.

The FTIR spectra of the pure CS/PVOH film (see line (1) in Figure 2) is a combination of both the CS and PVOH reflections. The large band at 3443 cm^−1^ is assigned to the stretching vibration of the hydroxyl groups of both CS and PVOH. The band at 3400 cm^−1^ is assigned the primary stretching vibration of the amino groups of CS. The same band is also assigned to the intra- and inter-molecular hydrogen bonds of the CS/PVOH matrix [16,33]. The band at 1637–1644 cm^−1^ is assigned to the associated water, to C–OH from the glycosidic units of the CS chains, and to the vibration of the carboxamide O= C–NHR of CS [16,34]. The band at 1150 cm^−1^ is assigned to the asymmetric bridge stretch of the glycosidic linkage of CS [16].

In the FTIR spectra of the CS/PVOH/xNZ films (see lines (2), (3), and (4) in Figure 2), the stretching reflections of NZ’s hydroxyl groups at 3740 cm^−1^, 3640, and 3540 cm^−1^ are also observed in addition to the presence of the CS/PVOH reflections. According to Tvaruskova and Bosacek [35], the band at 3740 cm^−1^ is independent of the degree of cationization of NZ. This band is attributed to the terminal hydroxyl group in the NZ crystal. The band at 3640 cm^−1^, denoted as a high-frequency (HF) band, is narrow and symmetrical, and its intensity depends on the degree of decationization. It was found that these hydroxyl groups are located in the large cavities of the Y zeolite; hence, they are easily accessible and can be affected by the sorption of saturated and unsaturated hydrocarbons [36]. The band at 3550 cm^−1^, denoted as a low-frequency (LF) band, is broad and asymmetrical, and it also depends on the degree of decationization. The hydroxyl groups corresponding to this band are located in the sodalite units of the zeolite structure and, although we assume that the protons in these groups are more loosely bound than those in the previous case, these hydroxyl groups are not sensitive with respect to the sorption of the nonpolar molecules or olefins because of their inaccessibility [35].

In the FTIR spectra of the CS/PVOH/xTO@NZ films (see lines (5), (6), and (7) in Figure 2), there are three main differences in comparison to the FTIR spectra of the CS/PVOH/xNZ films as follows: (i) the increase in the band at 3640 cm^−1^ of the NZ hydroxyl groups, (ii) the attenuation of the amino and hydroxyl group bands of the CS/PVOH matrix at 3400–3443 cm^−1^, and (iii) the attenuation of the band at 1640 cm^−1^ of the CS/PVOH matrix. The increase in the hydroxyl group band at 3640 cm^−1^ can be attributed to the adsorbed TO molecules on these sites according to the information mentioned hereabove about the ability of these hydroxyl group sites to adsorb saturated or unsaturated hydrocarbons. The attenuation of the bands at 3400–3443 cm^−1^ and 1640 cm^−1^ of the CS/PVOH matrix can be attributed to the interplay between the CS/PVOH matrix and the modified TO@NZ nanohybrid.

In any case, the FTIR spectra suggests the higher interplay and relaxation between the CS/PVOH matrix and modified TO@NZ nanohybrid than the CS/PVOH matrix and pure NZ. This result is in accordance with the XRD results shown hereabove and suggests a higher dispersion of the TO@NZ nanohybrid into the CS/PVOH matrix than the pure NZ.

### 2.3. Tensile Properties of CS/PVOH/xNZ and CS/PVOH/xTO@NZ Films

**Figure 3:**
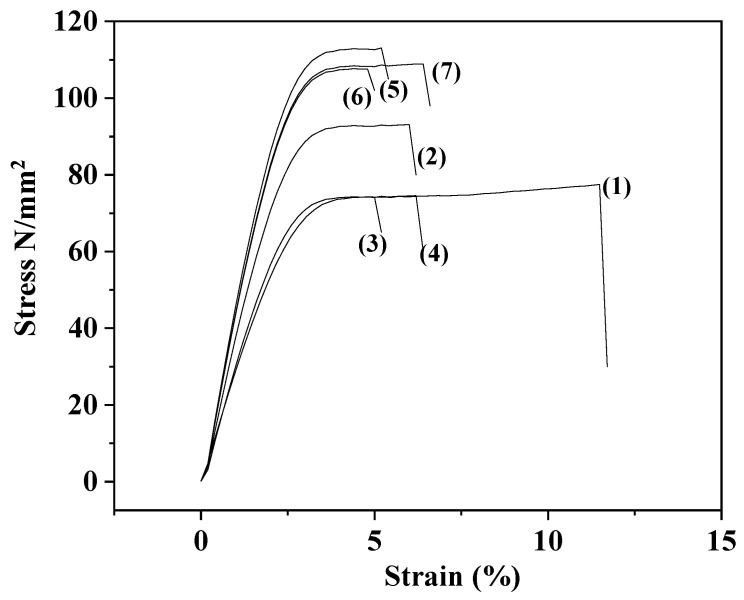
Stress–strain curves of (1) CS/PVOH, (2) CS/PVOH/5NZ, (3) CS/PVOH/10NZ, (4) CS/PVOH/15NZ, (5) CS/PVOH/5TO@NZ, (6) CS/PVOH/10TO@NZ, and (7) CS/PVOH/15TO@NZ obtained films.

In Figure 3, the representative stress–strain curves of all the CS/PVOH/xNZ and CS/PVOH/xTO@NZ films as well as the pure CS/PVOH film are shown for comparison. Using these stress–strain curves, Young’s (E) modulus, the ultimate tensile strength (σ_uts_), and the % strain at break (εb) were calculated and are listed in Table 1 for comparison.

From the values listed in Table 1 of Young’s (E) modulus, the ultimate tensile strength (σ_uts_), and the % strain at break (ε_b_), it is revealed that the addition of pure NZ in the CS/PVOH matrix increases the Young’s modulus values and decreases the elongation at break values. At the same time, the ultimate strength increases only in the case of the CS/PVOH/5NZ film and remains unchanged for the CS/PVOH/10NZ and CS/PVOH/15NZ films. This behavior is typical for rigid inorganic materials such as NZ blended with a polymer [37,38].

On the contrary, when the modified TO@NZ nanohybrid was added to the CS/PVOH matrix, both the stress and ultimate strength values increased, while the elongation at break values decreased. This behavior indicates that during the modification with the TO@NZ nanohybrids, the TO molecule acts as a kind of a compatibilizer and achieves a higher incorporation in the CS/PVOH matrix than the pure NZ. The results of the tensile properties, combined with the XRD analysis and FTIR spectrometry, show a higher dispersion and a higher relaxation of the TO@NZ nanohybrid with the CS/PVOH matrix compared to the relevant properties of the pure NZ, respectively.

### 2.4. Water–Oxygen Barrier Properties of CS/PVOH/xNZ and CS/PVOH/xTO@NZ Films

In Table 2, the obtained water–oxygen transmission rate values (WVTR) and the oxygen transmission rate (OTR) values for all the CS/PVOH/xNZ and CS/PVOH/xTO@NZ films as well as the pure CS/PVOH film are listed. Using the WVTR and OTR values, along with the film thickness, the water diffusivity (D_w_) and the oxygen permeability (PeO_2_) values were calculated and are listed in Table 2 for comparison.

By observing both the D_w_ and PeO_2_ values, it is obvious that the addition of both the pure NZ and modified TO@NZ nanohybrid reduces the water and oxygen permeability. As the wt.% content of the NZ and TO@NZ increase, the water and oxygen permeability further increase. A higher increase in the water–oxygen barrier was obtained for the TO@NZ-based samples than the pure NZ-based samples. Thus, both the pure NZ and modified TO@NZ are good barrier nanofillers, while the modified TO@NZ nanohybrid prevails to the pure NZ.

### 2.5. Total Antioxidant Activity of CS/PVOH/xNZ and CS/PVOH/xTO@NZ Films

The antioxidant activity of such active packaging films is a crucial parameter that extends the shelf life of foods and preserves their nutritional and aesthetic qualities by delaying the deterioration of food, which takes place through oxidation reactions.

**Figure 4:**
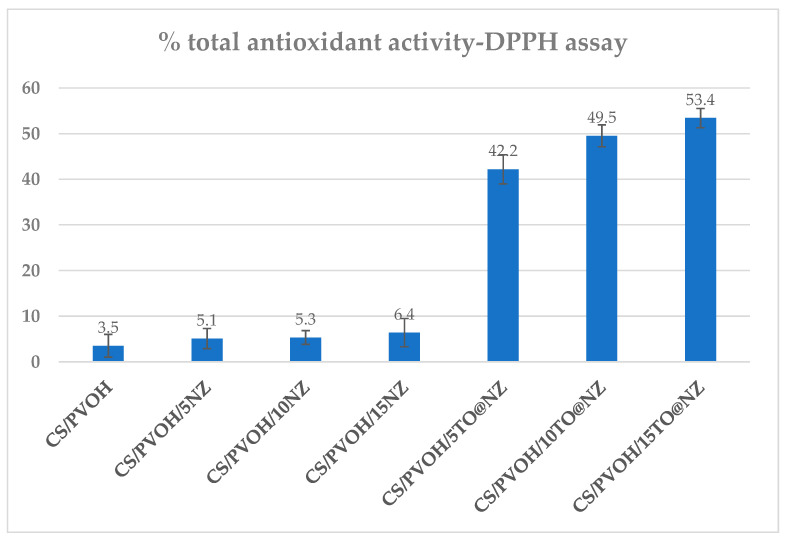
Calculated % of total antioxidant activity of all obtained CS/PVOH/xNZ and CS/PVOH/xTO@NZ films.

The calculated % of the total antioxidant activity values of all the CS/PVOH/xNZ and CS/PVOH/xTO@NZ films as well as the pure CS/PVOH film are plotted in Figure 4.

As expected, no significant antioxidant activity was obtained for the pure CS/PVOH and CS/PVOH/xNZ films. For the CS/PVOH/xTO@NZ films, the antioxidant activity increased as the TO@NZ wt.% content increased. The highest antioxidant activity value is equal to 53.4% and is obtained for the CS/PVOH/15TO@NZ film.

### 2.6. Antibacterial Properties of CS/PVOH/xNZ and CS/PVOH/xTO@NZ Films

**Figure 5:**
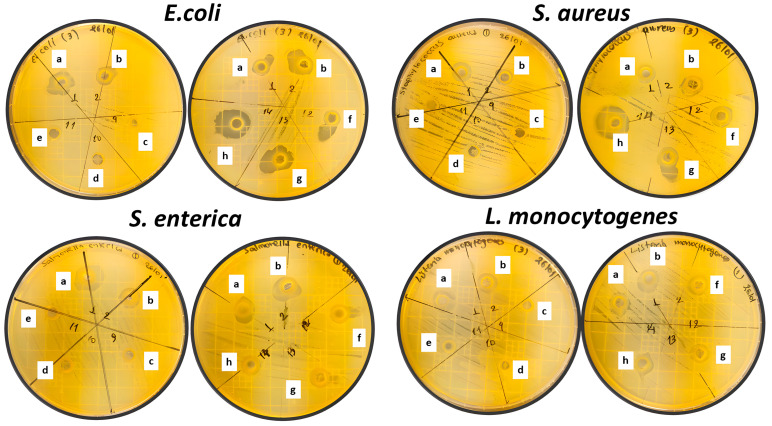
Petri dish images of (a) CS, (b) CS/PVOH, (c) CS20PVOH 5%NZ, (d) CS20PVOH 10%NZ, (e) CS20PVOH 15%NZ, (f) CS20PVOH 5%TO@NZ, (g) CS20PVOH 10%TO@NZ, and (h) CS20PVOH 15%TO@NZ films against *E. coli*, *S. aureus*, *S. enterica*, and *L. monocytogenes*.

The antibacterial efficacy of the investigated nano-reinforcement the CS/PVOH-based packaging films is presented in Table 3 and Figure 5.

The antibacterial activity of the different film materials was assessed against the following four foodborne pathogenic bacteria: *Escherichia Coli (E. coli)*, *Staphylococcus aureus (S. aureus)*, *Salmonella*. *Enterica (S. Enterica)*, and *Listeria monocytogenes (L. monocytogenes)*. The inhibitory activity of the film materials was assessed by measuring the diameter of the clear inhibition zone formed around the agar wells. In cases where no clear zone was observed surrounding the agar wells, it was interpreted as the absence of an inhibitory zone, and the diameter was recorded as zero.

The film material CS/PVOH displayed moderate antibacterial activity. Notably, the inhibition zone diameters against *E. coli and S. aureus* were 3.57 ± 0.55 and 4.23 ± 0.48, respectively, suggesting efficacy against these bacterial strains. The antimicrobial activity against *S. enterica* and *L. monocytogenes* were 3.26 ± 0.16 and 3.40 ± 0.70, respectively. This outcome was expected, since it is widely recognized that CS exhibits antibacterial activity, which can be attributed to the interaction between the positively charged ammonium (NH_4_^+^) groups present in the amino glucose units of CS and the negatively charged components of the bacterial cell wall. This interaction contributes to the antibacterial effects displayed by CS against various microorganisms [39]. In addition, as it was shown recently, the antibacterial activity of CS was supported and enhanced by the presence of PVOH [16,40].

Conversely, the film materials incorporating zeolite (CS/PVOH/NZ, CS/PVOH/10NZ, and CS/PVOH/15NZ) did not demonstrate observable inhibition zones against any of the tested bacteria. Furthermore, these three film materials were also subjected to testing of their antimicrobial activity using the disc diffusion method to investigate if they exhibited any activity when brought into direct contact with the inoculated agar surface. The findings reveal that despite the absence of an inhibition zone, there was evident antimicrobial activity observed at the contact area. The absence of the diffusion activity indicates that the antimicrobial compounds present in the films did not effectively spread any more throughout the surrounding medium when zeolite was added. Therefore, the inhibitory effect was primarily localized to the immediate vicinity of the films. Further research is warranted to investigate the factors influencing the diffusion capability of the antimicrobial compounds and to optimize the film formulation for enhanced antimicrobial activity.

On the other hand, all the tested materials containing thyme oil encapsulated with zeolite (CS/PVOH/5TO@NZ, CS/PVOH/10TO@NZ, and CS/PVOH/15TO@NZ) exhibited notable antimicrobial activity. Against *E. coli*, the resulting inhibition zone diameters were 3.93 ± 0.53, 5.35 ± 0.30, and 8.35 ± 0.45, respectively. Similarly, for *S. aureus*, the inhibition zone diameters were 4.73 ± 0.15, 5.32 ± 0.19, and 7.93 ± 0.54. These findings suggest that the inclusion of thyme oil encapsulated with zeolite enhanced the antimicrobial effectiveness of the film materials against the four mentioned kinds of microbes, and particularly against *S. aureus* and *E. coli*.

In summary, the film materials incorporating thymol encapsulated with zeolite demonstrated the highest antimicrobial activity. Conversely, the film materials containing zeolite alone did not exhibit significant antimicrobial efficacy. This finding aligns with those in the existing literature, which reports the lack of inherent antibacterial activity exhibited by zeolite itself [41,42].

Zeolite is characterized by its distinctive framework structure, consisting of interconnected channels and cavities. Within this framework, exchangeable cations help to maintain a balance by compensating for the permanent negative charge resulting from isomorphous substitution. The porous nature of zeolites enables them to adsorb water molecules, providing hydration to the exchangeable cations located within the framework. One of the remarkable properties of zeolites is their exceptional sorption capacity. They can accumulate various compounds, including water and salts. This sorption capability allows for zeolites to serve as carriers for active substances, such as antibacterial and antifungal compounds. These active compounds can be incorporated into the zeolite structure, taking advantage of the porous framework for controlled release over time [43].

In the present study, the variation in antimicrobial activity observed among the tested bacteria and the different films can be attributed mostly to the amount of loaded bioactive compound (thyme oil) onto film materials. It is well documented that higher loadings of essential oils result in stronger antimicrobial activity.

Comparable results were observed in another study, where thymol-impregnated starch-chitosan-zeolite films with thymol concentrations of 24% and 27% exhibited significant antibacterial effects against *S. aureus* and *E. coli*. This finding indicates that thymol was successfully released from the film into the surrounding culture medium while maintaining its antibacterial activity after impregnation within the polymer matrix [44].

Similarly, Pajnik et al. (2022) [42] reported analogous findings regarding the antibacterial effects of zeolite/chitosan/gelatin films. They observed that the antibacterial effect of the films alone, without thymol or carvacrol impregnation, was insignificant. However, when thymol or carvacrol was incorporated into the films, strong antibacterial activity was observed against both bacterial strains [42].

The antibacterial mechanism of phenolic compounds like TO is associated with their ability to disrupt the cell wall and membranes of bacteria. This disruption can lead to cell lysis and the release of cellular contents. Thymol, in particular, is known to integrate with the polar head groups of the lipid bilayer, inducing alterations in the cell wall [45].

Moreover, the antimicrobial activity of zeolites can be influenced by various factors such as the type, size, structure, physical appearance, the nature of the incorporated compound, and the loading concentration of the zeolite. Additionally, the specific microorganisms targeted and the environmental conditions can also affect the effectiveness of zeolites as antimicrobial agents. Therefore, it is important to consider these factors when utilizing zeolites for antimicrobial applications [46].

The combination of zeolites’ unique framework structure, exchangeable cations, and porous nature allows for the effective adsorption and release of active substances. This, in conjunction with the antimicrobial properties of active substances, allow for zeolites to exert their antimicrobial effects. The encapsulation of essential oils (EOs) in zeolites offers advantages in terms of physical stability, reduced volatility, and protection against light, humidity, and pH variations. This encapsulation also enables the controlled release of EOs under suitable conditions. Zeolites’ properties, such as their crystal size, morphology, porosity, and chemical composition, contribute to the successful encapsulation of EOs. Furthermore, zeolites exhibit good biocompatibility, low toxicity, and enhanced access to the micropores, making them suitable carriers for EO compounds. However, due to the high volatility, low photostability, and thermolability of EOs, the encapsulation in zeolites often may require the use of high-concentration solutions in order to exhibit a significant activity [47].

Overall, zeolites have demonstrated promising antimicrobial activity and hold potential for various applications in healthcare, environmental, and food safety sectors. Further research and development are ongoing to explore and optimize the antimicrobial properties of zeolites and their practical applications. These findings contribute valuable insights toward the development of antimicrobial film materials for potential applications in food packaging and related industries.

### 2.7. Visual Evaluation of the Antimicrobial Activity of the Active Pads against Mold of Fungi on Strawberries

Representative photos of packed strawberries inside the uncoated PP boxes (blank sample) and PP boxes coated with CS/PVOH, CS/PVOH/15NZ, and CS/PVOH/15TO@NZ hydrogels from days 0 to 21 are depicted in Table 4.

After 4 days of storage on the strawberries inside the uncoated box (blank sample) the growth of mold fungi is observed. In the case of strawberries inside the boxes coated with CS/PVOH and CS/PVOH/15NZ hydrogels, the growth of mold fungi is observed in the seventh and ninth days, respectively. On the contrary, in the case of strawberries inside the PP box coated with the CS/PVOH/15TO@NZ hydrogels, no mold fungi are observed until the 21st day of storage. This result is probably due to the CS/PVOH and CS/PVOH/15NZ hydrogels coated in the bottom side of the PP boxes, which probably succeeded in protecting the strawberries from mold fungi growth. The extension of life duration was confirmed only by visible observations, and it was around four days longer compared to the respective uncoated blank PP box. In addition to this result, the CS/PVOH/15TO@NZ active hydrogel succeeded to extend the protection of strawberries against mold fungi growth for three weeks. This result is very promising and shows that the CS/PVOH/15TO@NZ active hydrogel could be potentially applied as an active pad inside the bottom side of fruit and vegetable boxes and to extend their preservation time. It seems that the CS/PVOH/15TO@NZ active pad can protect fruit and vegetables due to its antibacterial properties and by releasing TO molecules inside the atmosphere of packaged fruit and vegetables as in the case of the strawberries.

## 3. Conclusions

According to the results of the above-mentioned analytical methods, the biopolymeric matrix, originated by the incorporation of the biodegradable byproduct chitosan and the biodegradable biopolymer polyvinyl alcohol, could be transformed to a promising food packaging material by the addition of TO@NZ nanohybrid material. The overall study indicates that the addition of 15% TO@NZ nanohybrid material to the CS/PVOH biopolymeric matrix led to a potential food packaging film with a 34% higher Young’s modulus (E) and a 46.5% higher strength (σ_uts_) compared to the relevant properties of the pure CS/PVOH film. The only measurable drawback was the elasticity of the film, which is represented by the elongation at the break property. This property was reduced by −66%. All previous properties became worse while the pure NZ was added to the same CS/PVOH biopolymeric matrix. The XRD and FTIR measurements indicated that the pure NZ could not be incorporated well with the CS/PVOH biopolymeric matrix and created aggregations inside the film. On the contrary, the same techniques indicated that the TO@NZ nanostructures were well incorporated with the CS/PVOH polymeric matrix, and a homogeneous film with no aggregation phenomena was developed. This achievement is due to the enforcement of dispersion offered by TO to the film.

The overall conclusion is that the CS/PVOH/15TO@NZ film is the optimum developed material that exhibits a higher antioxidant activity, i.e., 53.4% higher than the pure CS/PVOH, and a higher antimicrobial activity, i.e., 134% for the *E. coli*, 87.5% for the *S. aureus*, 9% for the *S. enterica*, and 32% for the *L. monocytogenes*. The water vapor diffusion coefficient and the oxygen permeability coefficient decreased to −51% and −74%, respectively, and thus, the water vapor and oxygen barrier increased. Such results are obvious by observing the in vivo tests performed on the strawberries, where in the cases of CS/PVOH or CS/PVOH/xNZ, the strawberries were rotted after 9 days, while the strawberries packaged with CS/PVOH/15TO@NZ rotted after 21 days. This resulted because of the modified atmosphere by the controlled release TO.

## 4. Materials and Methods

### 4.1. Materials

Chitosan (CS) with a molecular weight of 100,000–300,000 was purchased from Acros Organics company (Zeel West Zone 2, Janssen Pharmaceuticalan 3a, B2440, Geel, Belgium). Polyvinyl alcohol, 86–89% hydrolyzed, low molecular weight, was purchased from Thermo Scientific Chemicals Co., (168 Third Avenue, Waltham, Massachusetts MA, 02451, USA). Edible activated natural zeolite was purchased from a local pharmacy market (≤20 μm, ≥650 m^2^/g, Serbian Micronized Zeolite, Product code HTSF257, Supplier Health Trade Athens, Greece). The used thyme oil was produced by the Chemco company (Via Achille Grandi, 13–13/A, 42030 Vezzanosul, Crostolo, Italy), and acetic acid (CAS Number: 64-19-7) was supplied by Sigma-Aldrich (Co., 3050 Spruce Street, St. Louis, Missouri, MO, 314-771-5765, USA).

### 4.2. Modification of NZ with Thymol

The modification of NZ that was rich in thymol cluster took place according to previous reports [24,48]. Briefly, thyme oil was first distillated at 200 °C to remove the cluster of D-Limonene and p-Cymene. The remaining thymol oil cluster (TO) was evaporated at 250 °C in a handmade apparatus in the upper part of which 3 g of NZ bed was adjusted. Differential scanning calorimetry showed that thymol was the main cluster of molecules adsorbed on NZ, while the adsorption of TO molecules was physiosorbed rather than chemisorbed [25]. Thermogravimetric analysis experiments of NZ and TO@NZ showed that the wt.% amount of TO adsorbed was 35.5% [25]. The obtained TO@NZ nanohybrid was further stored to be used in the preparation of films.

### 4.3. Preparation of CS/PVOH/xNZ and CS/PVOH/xTO@NZ Films

**Figure 6:**
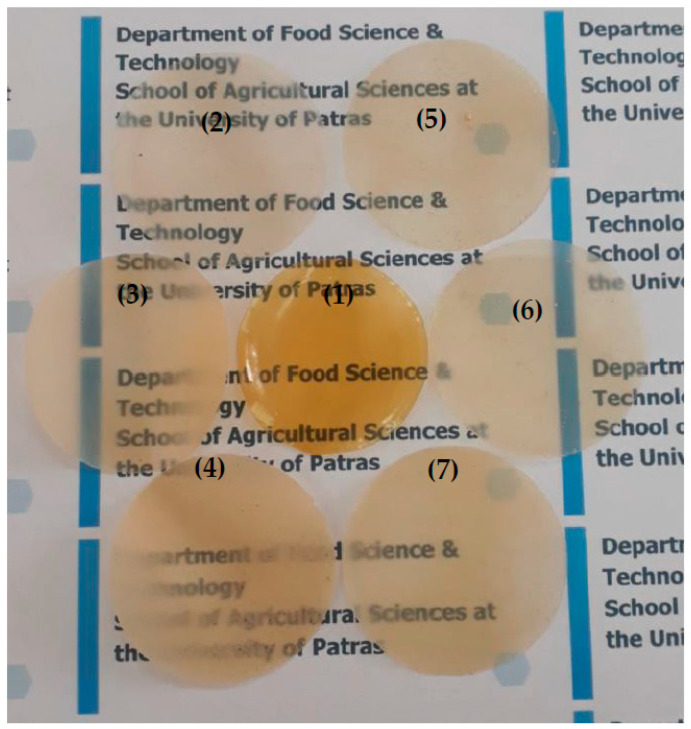
Photo of all obtained films. (1) Pure CS/PVOH, (2) CS/PVOH/5NZ, (3) CS/PVOH/10NZ, (4) CS/PVOH/15NZ, (5) CS/PVOH/5TO@NZ, (6) CS/PVOH/10TO@NZ, and (7) CS/PVOH/15TO@NZ.

First, an acetic acid (1% *v*/*v*) with 2 wt.% CS aqueous solution was prepared by adding 20 g of CS powder in 990 mL of distilled water and 10 mL of glacial acetic acid. The solution was heated at 70 °C and stirred overnight until a homogenous hydrogel free of bubbles was obtained. Second, 12 g of PVOH was added in 120 mL of distilled water, stirred, and heated until diluted to obtain a 10 wt.% PVOH aqueous solution. For each film, 90 mL of acetic acid (1% *v*/*v*) with 2 wt.% CS aqueous solution was mixed with 12 mL of 10 wt.% PVOH aqueous solution to obtain a 30 wt.% PVOH nominal content. In this CS/PVOH solution, 0.21 g, 0.44 g, and 0.71 g of NZ or TO@NZ powder was added to achieve 5, 10, and 15 wt.% NZ and TO@NZ final nominal content. The obtained mixtures were homogenized at 18.000 rpm for 5 min to obtain CS/PVOH/xNZ and CS/PVOH/xTO@NZ hydrogels (where x = 5, 10, and 15). The obtained hydrogels were spread out on plastic Petri dishes with 11 cm diameter and were dried at 25 °C to evaporate water and obtain the final CS/PVOH/xNZ and CS/PVOH/xTO@NZ films. The obtained films were peeled off from the Petri dishes and stored at 25 °C and 50% RH for further use and characterization. For comparison, a CS/PVOH film without the addition of NZ or TO@NZ powder was made and was considered as the “blank” sample. In Figure 6, images of all obtained CS/PVOH/xNZ and CS/PVOH/xTO@NZ films as well as the pure CS/PVOH film are shown. In all cases, high transparency was obtained, indicating a good dispersion of both pure NZ and TO@NZ nanohybrid in CS/PVOH/xNZ and CS/PVOH/xTO@NZ hydrogels.

### 4.4. XRD Analysis of CS/PVOH/xNZ and CS/PVOH/xTO@NZ Films

For XRD measurements of each film, a Brüker D8 Advance X-ray diffractometer instrument (Brüker, Analytical Instruments, S.A., Athens, Greece) was employed. A small piece of each film was attached to the sampler of the instrument, and the measurements were carried out in the range 2θ = 0.5–30° and with an increment of 0.03°.

### 4.5. FTIR Spectroscopy of CS/PVOH/xNZ and CS/PVOH/xTO@NZ Films

For the FTIR spectroscopy measurements, an FT/IR-6000 JASCO Fourier transform spectrometer (JASCO, Interlab, S.A., Athens, Greece) was employed. Prior to measurements, CS/PVOH, CS/PVOH/xNZ, and CS/PVOH/xTO@NZ films were cut in very small pieces and incorporated to the KBr used for the measurements. The measurements were carried out in the range of 4000–400 cm^−1^, with a 2 cm^−1^ resolution, and 64 scans for the final mean value of each point. An initial background was measured under the same conditions using pure KBr. Y-axis of all FT-IR measurements was the % transmittance.

### 4.6. Tensile Measurements of CS/PVOH/xNZ and CS/PVOH/xTO@NZ Films

Tensile properties of obtained CS/PVOH, CS/PVOH/xNZ, and CS/PVOH/xTO@NZ films were estimated according to the ASTM D638 method by using a Simantzü AX g 5kNt instrument (Simandzu Asteriadis, S.A., Athens, Greece). This ASTM method imposes the use of type V “dog bone” samples with dimensions 0.22 mm × 3.1 mm × 15 mm.

### 4.7. Water Vapor Transmission Rate Measurements and Water Diffusion Coefficient Calculation

The water vapor transmission rate (WVTR g/cm^2^ s) for all obtained CS/PVOH, CS/PVOH/xNZ, and CS/PVOH/xTO@NZ films was measured at 38 °C and 95% RH using a handmade apparatus and employing the ASTM E96/E 96M-05 method. The WVTR values were calculated and transformed to water vapor diffusivity (D_wv_) values according to the theory and equations described in detail in previous publications [39,47].

### 4.8. Oxygen Transmission Rate Measurements and Oxygen Permeability Calculation

Oxygen transmission rate (OTR) values (cc O_2_/m^2^/day) for all obtained CS/PVOH, CS/PVOH/xNZ, and CS/PVOH/xTO@NZ films were measured by employing an oxygen permeation analyzer (O.P.A., 8001, Systech Illinois Instruments Co., Johnsburg, IL, USA) at 23 °C and 0% RH according to the ASTM D 3985 method. The oxygen permeability coefficient values (PeO_2_) were calculated according to the theory and equations provided in detail in previous publications [39,47].

### 4.9. Total Antioxidant Activity of CS/PVOH/xNZ and CS/PVOH/xTO@NZ Films

The total antioxidant activity of all obtained CS/PVOH/xNZ and CS/PVOH/xTO@NZ films was evaluated according to diphenyl-1-picrylhydrazyl (DPPH) assay [37]. Briefly, 300 mg of each film was placed inside dark glass bottles with 10 mL of a 40 ppm ethanolic solution of DPPH. The absorbance at 517 nm wavelength of the DPPH solution was measured at 0 h and after 24 h of incubation using a Jasco V-530 UV-Vis spectrophotometer. For comparison, the absorbance of a 10 mL of ethanolic DPPH solution without the addition of any film was measured at 517 nm and considered as the blank sample for each kind of film, three different samples were measured, and the statistical mean was achieved as the final measurement.

The % of antioxidant activity after 24 h of incubation of films was calculated according to the following equation:

% Antioxidant activity = (Abs_blank_ − Abs_sample_)/Abs_blank_ × 100 (1)

### 4.10. Antibacterial Activity Tests of CS/PVOH/xNZ and CS/PVOH/xTO@NZ Films

Antimicrobial activity of the films was investigated using the well diffusion method against four foodborne pathogenic bacteria. The bacteria tested included *Escherichia coli* (ATCC 25922) and *Salmonella enterica* subsp. *enterica* (DSMZ 17420), which are Gram-negative bacteria, as well as *Staphylococcus aureus* (DSMZ 12463) and *Listeria monocytogenes* (DSMZ 27575), which are Gram-positive bacteria. These bacterial strains were obtained from the Institute of Technology of Agricultural Products, ELGO-DEMETER, located in Lykovryssi, Greece. Initially, the bacterial strains were cultured in Mueller–Hinton broth at a temperature of 37 °C for a duration of 24 h to allow for growth and to achieve a bacterial concentration ranging from 10^7^ to 10^8^ of colony-forming units per milliliter (CFU mL^−1^). Following the overnight incubation, the bacteria were evenly distributed on Mueller–Hinton agar plates by rotating the plates at 60-degree intervals to ensure uniform growth of the bacterial colonies. Plates inoculated with the test organism had 6 mm wells cut into the surface of the agar using a cork borer dipped in alcohol and flamed. The wells were filled with 100 μL of the studied suspensions with which the final films were formed. The plates were then incubated at a temperature of 37 °C overnight. After incubation, the diameters of any clear zones around the antimicrobial-containing wells were measured using calipers. This measurement was conducted to assess the extent of antimicrobial activity exhibited by the films against the tested bacteria. The entire experiment was performed in triplicate to ensure reliable and consistent results.

### 4.11. Packaging Test of CS/PVOH/HNT- and CS/PVOH/TO@HNT-Based Active Pads in Strawberry Protection against the Mold Fungi

Considering the extensive research reported in the literature wherein CS/EO hydrogels were implemented as a protective coating for the preservation of strawberries, a novel methodology/technology for strawberry preservation was devised. This method was preconceived the most efficacious CS/PVOH/15TO@NZ sample as an active pad, positioned at the base of a PP plastic fruit container. The goal of this approach was the modification of the atmosphere inside the fruit container through the controlled release of TO from packaging material. In detail, 50 mL of CS/PVOH, CS/PVOH/xNZ, and CS/PVOH/xTO@NZ solutions were spread in the bottom side of the PP fruit boxes and were left to dry to make an active pad inside the PP box. Strawberries that were as close to the same shape as possible and with the same ripeness were purchased from the local supermarket, washed with water, and divided into four groups with three fruits each. In each box of PP coated with CS/PVOH, CS/PVOH/xNZ, and CS/PVOH/xTO@NZ solution, three strawberries were included, and the box was closed and kept under room temperature conditions (10–20 °C and 50% RH). For comparison, three strawberries were put inside an uncoated PP box, which named as the blank sample. The strawberries in the blank PP box as well as the strawberries inside the CS/PVOH-, CS/PVOH/xNZ-, and CS/PVOH/xTO@NZ-coated PP boxes were monitored with daily photos for 21 days to investigate possible growth of mold fungi.

### 4.12. Statistical Analysis

The statistical software IBM, SPSS ver. 25, was used to treat the resulting measurements of three pieces of every film sample for each one property presented in Table 1, Table 2 and Table 3. Values presented in these tables are the final mean values of these three measurements for every property, and the plus/minus (±) standard deviation is tabulated on the right of each value. A confidence interval of C.I. = 95% was assumed in every case. Hypothesis tests assuming a statistical significance level of *p* = 0.05 was carried out for every case to ensure that different mean values of a property for different samples are also statistically different. The non-positive normality tests implied the non-parametric Kruskal–Wallis method for such investigations, and statistically equal mean values are indicated in tables with the same superscript index.

## Figures and Tables

**Table 1:** Calculated values of Young’s (E) modulus, ultimate tensile strength (σ_uts_), and % strain at break (ε_b_).

	E	σ_uts_	ε%
CS/PVOH	2249.3 ± 200.3	71.2 ± 1.8 ^c^	11.8 ± 0.9
CS/PVOH/5NZ	3064.3 ± 26.3 ^a^	89.0 ± 4.6	6.9 ± 1.1 ^e,f^
CS/PVOH/10NZ	2736.0 ± 351.3 ^b^	71.0 ± 16.7 ^c^	6.7 ± 2.8 ^f^
CS/PVOH/15NZ	2803.4 ± 345.3 ^b^	73.3 ± 4.5 ^c^	7.0 ± 2.1 ^e^
CS/PVOH/5TO@NZ	3304.0 ± 279.5	109.3 ± 17.2	6.8 ± 1.2 ^f^
CS/PVOH/10TO@NZ	3186.5 ± 125.2	103.7 ± 1.4 ^d^	6.7 ± 2.3 ^f^
CS/PVOH/15TO@NZ	3010.0 ± 481.3 ^a^	104.7 ± 5.5 ^d^	7.1 ± 2.2 ^e^

Results are expressed as mean ± standard deviation (n = 3). Means in the same column bearing same superscript letters, i.e., a, b, c, d, e, f, are significantly equal (*p* < 0.5).

**Table 2:** Film thickness, water vapor transmission rate (WVTR), water diffusivity (D_W_), oxygen transmission rate (OTR), and oxygen permeability (PeO_2_) values of pure CS/PVOH film as well as of CS/PVOH/HNT and CS/PVOH/TO@HNT films.

	Film Thickness (mm)	Water Vapor Transmission Rate (10^−6^ g/cm^2^·day)	D_w_—Water Diffusion Coefficient (10^−4^ cm^2^/s)	Oxygen Transmission Rate (mL/m^2^·day)	Pe_O2_ —Oxygen Permeability (10^−7^ cm^2^/s)
CS/PVOH	0.17 ± 0.012	1.06 ± 0.12	3.65 ± 0.11	38.2 ± 0.2	6.5 ± 0.3
CS/PVOH/5NZ	0.11 ± 0.010 ^a^	1.17 ± 0.10	3.21 ± 0.09	33.7 ± 0.2	3.7 ± 0.1
CS/PVOH/10NZ	0.12 ± 0.009	1.01 ± 0.13	2.67 ± 0.12	26.1 ± 0.2	2.6 ± 0.1
CS/PVOH/15NZ	0.10 ± 0.005 ^b^	0.92 ± 0.09 ^a^	2.33 ± 0.08	27.5 ± 0.3	2.2 ± 0.2
CS/PVOH/5TO@NZ	0.11 ± 0.006 ^a^	0.92 ± 0.07 ^a^	2.03 ± 0.06	15.5 ± 0.2 ^a^	1.8 ± 0.2 ^a^
CS/PVOH/10TO@NZ	0.08 ± 0.003	0.81 ± 0.07 ^b^	1.6 ± 0.05	34.4 ± 0.2	3.4 ± 0.1
CS/PVOH/15TO@NZ	0.10 ± 0.009 ^b^	0.79 ± 0.05 ^b^	1.8 ± 0.04	15.0 ± 0.2 ^a^	1.7 ± 0.2 ^a^

Results are expressed as mean ± standard deviation (n = 3). Means in the same column bearing same superscript letters, i.e., a, b, are significantly equal (*p* < 0.5).

**Table 3:** Antimicrobial activity of active films against food pathogenic bacteria *E. coli*, *S. aureus*, *S. enterica*, and *L. monocytogenes*.

Film Material	*E. coli*	*S. aureus*	*S. enterica*	*L. monocytogenes*
	Inhibition (Diameter of Clear Zone)	Inhibition (Diameter of Clear Zone)	Inhibition (Diameter of Clear Zone)	Inhibition (Diameter of Clear Zone)
CSPVOH	3.57 ± 0.55 ^a^	4.23 ± 0.48	3.26 ± 0.17 ^a^	3.40 ± 0.70 ^a^
CSPVOH5NZ	0.00 ^b^	0.00	0.00 ^b^	0.00 ^b^
CSPVOH10NZ	0.00 ^b^	0.00	0.00 ^b^	0.00 ^b^
CSPVOH15NZ	0.00 ^b^	0.00	0.00 ^b^	0.00 ^b^
CSPVOH5%TO@NZ	3.93 ± 0.53 ^a^	4.73 ± 0.15	3.37 ± 0.16 ^a^	3.70 ± 0.14 ^a^
CSPVOH10TO@NZ	5.35 ± 0.30	5.32 ± 0.19	3.53 ± 0.18 ^c^	4.05 ± 0.18
CSPVOH15TO@NZ	8.35 ± 0.45	7.93 ± 0.54	3.55 ± 0.07 ^c^	4.48 ± 0.08

Inhibitory zone surrounding film discs measured in mm after the subtraction of the well diameter (6 mm). Results are expressed as mean ± standard deviation (n = 3). Means in the same column bearing the same superscript letters, i.e., a, b, c, are significantly equal (*p* < 0.5).

**Table 4:** Photos of packed strawberries inside the uncoated PP boxes (blank sample) and the PP boxes coated with CS/PVOH, CS/PVOH/15NZ, and CS/PVOH/15TO@NZ hydrogels from days 0 to 21.

	Day 0	Day 2	Day 4	Day 6
BLANK	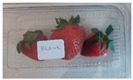	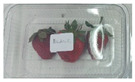	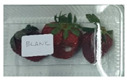	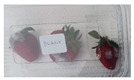
CS/PVOH	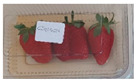	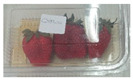	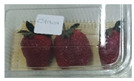	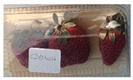
CS/PVOH/15NZ	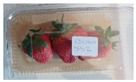	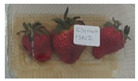	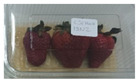	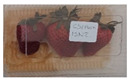
CS/PVOH/15TO@NZ	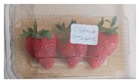	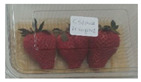	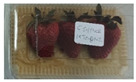	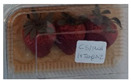
	Day 7	Day 9	Day 10	Day 12
CS/PVOH	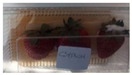	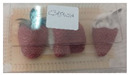		
CS/PVOH/15NZ	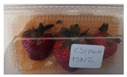	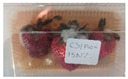		
CS/PVOH/15TO@NZ	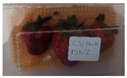	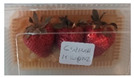	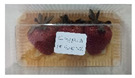	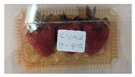
	Day 14	Day 16	Day 18	Day 21
CS/PVOH/15TO@NZ	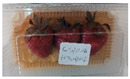	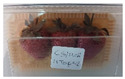	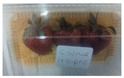	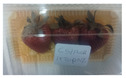

## Data Availability

The datasets generated for this study are available upon request to the corresponding authors.
